# Adult granulosa cell tumour of the ovary incidentally discovered during caesarean section in a pregnant patient after IVF: a rare case and a review of the literature

**DOI:** 10.3332/ecancer.2023.1496

**Published:** 2023-01-09

**Authors:** Kong Chi Pham, Phuong Thi Minh Ta, Nhat Minh Huynh

**Affiliations:** 1 Department of Gynecology, Da Nang Hospital for Women and Children, 402 Le Van Hien Street, Ngu Hanh Son District, Da Nang City 550000, Vietnam; 2 Department of Pathology, Da Nang Hospital for Women and Children, 402 Le Van Hien Street, Ngu Hanh Son District, Da Nang City 550000, Vietnam

**Keywords:** Foetal outcome, granulosa cell tumour, in vitro fertilisation, maternal outcome, pregnancy

## Abstract

Adult granulosa cell tumours (AGCTs) of the ovary are very rare during pregnancy. To date, only five cases of ovarian AGCT in pregnancy have been reported in the literature and the patients all conceived spontaneously. We report a case of AGCT of the ovary that was incidentally discovered during a caesarean section in a patient undergoing In vitro fertilisation (IVF). To the authors’ knowledge, this is the first case of AGCT incidentally discovered during caesarean section in a pregnant patient after IVF. A 44-year-old primigravida with 39 weeks gestation was admitted to our hospital due to premature rupture of membranes in May 2019. She was treated by *in vitro* fertilisation due to being an elderly mother and she was pregnant after the first cycle. She was indicated for caesarean section due to conceiving following *in vitro* fertilisation and being an elderly mother. She gave birth to a 3,000 g baby boy and his Apgar scores were 8/1^’^–9/5^’^. When examining the adnexa, the left ovary had a tumour with a size of 7 × 4 × 4 cm. Left oophorectomy was performed and specimen sent to for histopathology. The histopathological diagnosis was an AGCT of the ovary. A month later, the patient received chemotherapy with Carboplatin and Paclitaxel for four cycles. After 32 months of follow-up, no recurrence was detected. In conclusion, AGCTs of the ovary are very rare during pregnancy. Pre-operative diagnosis is difficult. Conservative surgery should be considered in women who wish to have children. Patients should receive adequate counselling and long-term follow-up to ensure the highest survival rates and early detection of recurrence.

## Introduction

Granulosa cell tumour of the ovary is a rare malignancy, originating from ovarian sex cord stromal cells. The rate of ovarian granulosa cell tumour is 0.2/100,000 women, accounting for only 2%–5% of all malignant ovarian tumour cases. Based on clinical and histological features, ovarian granulosa cell tumours are divided into two types: adult (95%) and juvenile (5%) [1].

Ovarian granulosa cell tumour is very rare during pregnancy. The systematic review by Blake *et al* [2] showed that there were only 46 cases of ovarian sex cord stromal tumours in pregnancy reported in the literature from 1950 to 2012, of which 13 cases were granulosa cell tumour. According to Guidi’s *et al* [3] review published in 2021, only five cases of adult granulosa cell tumour (AGCT) of the ovary during pregnancy were reported in the literature and the patients all conceived spontaneously.

Clinical and subclinical features of AGCT are often nonspecific, with 1/5 of cases asymptomatic and incidentally detected [4]. Due to the rarity of the disease, consensus on the best treatment has not yet been reached. Surgery remains the gold standard in the treatment of AGCT of the ovary. The role of lymphadenectomy and chemotherapy has not been clearly defined [5]. The prognosis of granulosa cell tumour of the ovary is usually good, but the recurrence rate is high (36%); therefore, long-term follow-up is important. Disease stage is a significant prognostic factor for recurrence [6]. The relationship between ovarian stimulation in the treatment of infertility and ovarian cancer as well as granulosa cell tumour of the ovary has not been clearly defined [7, 8].

We report a case of an AGCT of the ovary that was incidentally discovered during a caesarean section in a patient undergoing In vitro fertilisation (IVF). To the authors’ knowledge, this is the first case of AGCT incidentally discovered during caesarean section in a pregnant patient after IVF One month later, the patient received chemotherapy with Carboplatin and Paclitaxel for four cycles and was re-examined according to the regimen. After 32 months of follow-up, no clinical or subclinical recurrence was detected.

## Case report

**Figure 1.**
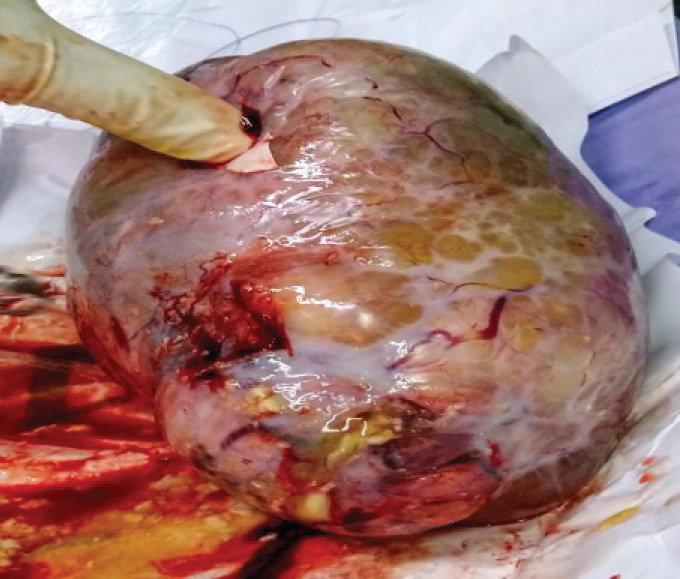
Mass of left ovary with smooth capsule

A 44-year-old primigravida with 39 weeks gestation was admitted to our hospital due to premature rupture of membranes in May 2019. In her past obstetrical and gynaecological history, she had primary infertility for 3 years. Tests to investigate the cause of infertility for both the wife (basic endocrine including Follicle Stimulating Hormone (FSH), Luteinizing Hormone (LH), oestrogen, testosterone, prolactin, anti-Mullerian hormone (AMH), gynaecological ultrasound and hysterosalpingography) and husband (endocrine and semen analysis) were normal. She was treated by *in vitro* fertilisation due to being an elderly mother and she was pregnant after the first cycle. Antenatal care in the first and second trimester at private hospitals did not detect any abnormality: fetal abnormality, and maternal status (internal and surgical medical history, uterus and ovaries problems). She was indicated for caesarean section due to conceiving following *in vitro* fertilisation and being an elderly mother. She gave birth to a 3,000 g baby boy and his Apgar scores were 8/1^’^–9/5^’^. When examining the adnexa, the left ovary had a tumour with a size of 7 × 4 × 4 cm, and the capsule was intact. Tumour was rough inside, hard shell removed (Figure 1). The right adnexa were normal. Peritoneum and omentum were also normal. Left oophorectomy was performed and specimen sent to for histopathology. We discussed with pathologist from Ho Chi Minh City Oncology Hospital – one of the largest Oncology hospital in Vietnam.

**Figure 2.**
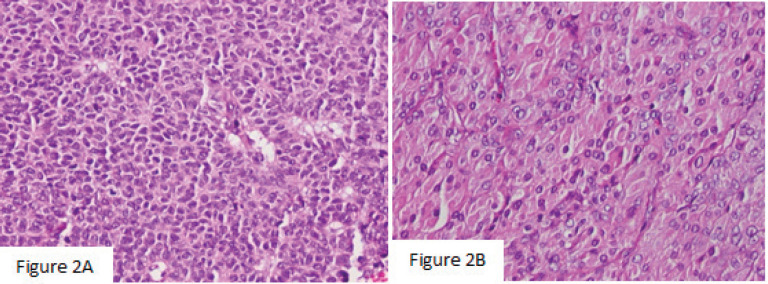
(a): The histology showed that the cells were arranged in various patterns, including diffuse, trabecular and corded, insular, microfollicular, Call-Exner Bodies (×200). (b): Luteinised cells with moderate to abundant eosinophilic cytoplasm, conspicuous nucleoli, no nuclear grooves (×400).

**Figure 3.**
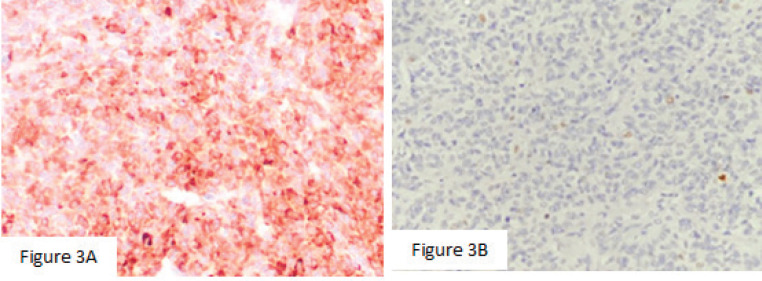
(a): Mitotic activity was 4/10 high power fields. (b): Immunohistochemistry was positive for inhibin (×400), negative for EMA (×200).

Macroscopically, the left ovarian tumour had a smooth surface, measured at 8 cm in its greatest diameter and was well demarcated. On the cut surface, it appeared yellowish solid, cystic, soft and focally haemorrhagic, with necrosis. On microscopic examination, it was small, bland cells with scant cytoplasm and pale, uniform angulated and grooved nuclei (coffee bean). The cells were arranged in various patterns, including diffuse, trabecular and corded, insular, microfollicular, Call-Exner Bodies (Figure 2a). Luteinised cells with moderate to abundant eosinophilic cytoplasm, conspicuous nucleoli, no nuclear grooves (Figure 2b). Mitotic activity was 4/10 high power fields. Immunohistochemistry was positive for inhibin (Figure 3a), negative for Epithelial membrane antigen (EMA) (Figure 3b). The final diagnosis was an AGCT of the ovary.

Magnetic resonance image (MRI) for the pelvic abdomen after caesarean section did not detect any abnormal signals. The patient was stage Ia. We consulted with oncologists, and the given regimen was adjuvant chemotherapy (Carboplatin + Paclitaxel).

A month later, she received adjuvant chemotherapy with four cycles of carboplatin (300 mg/m^2^) on day 1 and paclitaxel (175 mg/m^2^) on day 1 every 3 weeks. After 32 months follow-up, no recurrence has been detected in clinical or laboratory situation as well as Magnetic Resonance Imaging (MRI).

The study was approved by the Ethical Review Committee and performed in accordance with the principles of the Declaration of Helsinki. The patient provided written informed consent for the publication and the use of her images.

## Discussion

AGCT of the ovary accounts for 95% of granulosa cell tumour cases and is usually diagnosed in the peri- and postmenopausal stage, while the less common juvenile type is usually diagnosed at first three decades of life [1]. Five cases of ovarian AGCT in pregnancy were reviewed by Guidi *et al* [3] adding one our case. We summarised circumstances and time of detection, foetal outcomes in Table 1 and management, maternal outcomes in Table 2. There were three cases discovered by chance during caesarean section, two cases recurred and one case was discovered at 15 weeks gestation. Three cases had no symptoms, one case had abdominal pain because of threatened preterm birth, one case had nonspecific abdominal symptoms and one recurred case had enlarged and palpable tumour.

Preoperative diagnosis of granulosa cell tumour is challenging for physicians. Clinical and laboratory features of granulosa cell tumour of the ovary are often nonspecific [4]. Granulosa cell tumour of the ovary has the ability to secrete oestrogen, causing symptoms such as abnormal vaginal bleeding in postmenopausal women and heavy menstrual bleeding in younger women. The most common clinical symptom was abnormal vaginal bleeding (44.6%), abdominal pain (25.8%) [12]. The International Ovarian Tumor Analysis gave two findings suggestive of granulosa cell tumour on ultrasound: (i) solid mass with heterogeneous echoes and (ii) polycystic solid mass with mixed or low echo, known as the ‘Swiss Cheese’ sign. However, because of the rarity of the disease, the value of these markers has not been determined. To date, only six reports have evaluated the sonographic features of granulosa cell tumour [13]. As for magnetic resonance, the reports are neither descriptive nor comprehensive in characterisation of granulosa cell tumour. Inhomogeneous signal intensity on both T1-weighted image (T1WI) and T2-weighted image (T2WI) and high signal intensity on Diffusion-weighted imaging (DWI) imaging are suggestive markers for the diagnosis of granulosa cell tumour of the ovary [14]. Today, the early detection of adult-type granulosa cell tumour of the ovary can be based on inhibin B and AMH tests. Because inhibin B levels fluctuate during the menstrual cycle and are also increased in some other ovarian cancers (e.g.: epithelial ovarian cancer), AMH is more specific for AGCT of the ovary [2, 3]. AMH has a high sensitivity (92%) and specificity (81%) in detecting macroscopic AGCT of the ovary. Combination of both hormones will have higher diagnostic value than inhibin alone [15]. Our patient had no specific symptoms and the tumour was discovered incidentally during caesarean section. According to Levin *et al* [4] 1/5 of AGCT of the ovary is asymptomatic and diagnosed incidentally, whereas most cases of juvenile granulosa cell tumour of the ovary are symptomatic at the time of diagnosis.

In the five cases of AGCT of the ovary reported above, the patients all conceived spontaneously. Meanwhile, our patient got pregnant after IVF treatment. Although AMH level and gynaecological ultrasound at the time of the infertility investigation as well as during ovarian stimulation and prenatal care screening for this patient did not detect any abnormalities, it is difficult to determine whether the granuloma was pre-existing or occurred during ovarian stimulation or during pregnancy. The relationship between the use of ovulation induction in infertility treatment and granulosa cell tumour has not been established. Several theories have been put forward to explain this association, such as (i) granulosa cell tumour is present in the ovary and when primed with hormones, they will grow, (ii) high levels of follicle-stimulating hormone oocytes have a carcinogenic effect on granulosa cells and (iii) the onset of granulosa cell tumour during ovarian stimulation is coincidental [8]. A recent Cochran library review (2019) found that infertility is an important risk factor for ovarian cancer. However, when evaluating the association between fertility drugs and ovarian cancer, other factors such as age, body mass index, genetic factors (e.g. family history of ovarian cancer) and causes of infertility should be considered [7].

Surgery remains the mainstay of treatment for primary or secondary AGCT including complete resection of the tumour, uterus, adnexa and staging procedures (peritoneal washings, biopsies and omentectomy). Routine dissection of pelvic and para-aortic lymph nodes is not recommended. Only large or suspicious lymph nodes are removed. For cases where the disease is localised (stage Ia) and where fertility is desired, the tumour adnexa can be removed, preserving the uterus and contralateral ovary. However, careful staging is required in these cases and endometrial biopsy is recommended to rule out metastases and/or concomitant endometrial disease. For early-stage cases, no additional medical treatments are needed. Chemotherapy may be indicated in advanced or inoperable cases, although efficacy and prognostic significance are uncertain. The role of adjuvant chemotherapy in AGCT remains controversial and does not appear to have a significant impact on patient outcomes [5].

Studies show that tumour size is related to disease prognosis, tumour size < 5 cm has a 10-year survival rate of 100%, this rate is 63% with size of 5–15 cm and >15 cm is 34%. Cells with atypical nuclei are considered the most reliable indicator in stage I. For later stages, in addition to atypical nuclei, the percentage of mitotic cells is a poor prognostic factor. However, the correlation between these two factors and the prognosis of the disease has not been determined yet [16]. Our patient had risk factors such as tumour size of 7 cm, histopathological findings with round or coffee bean-shaped nuclei, high number of atypical mitosis. In addition, the patient still wanted to preserve fertility, so the uterus and ovaries were kept. Therefore, this is an incomplete surgery. Based on the above factors, the patient received four cycles of adjuvant chemotherapy Carboplatin + Paclitaxel and was given a long-term follow-up. We stopped at the fourth chemotherapy cycle because of severe side effects – vomiting and neutropenia.

The prognosis of AGCT is generally good. The 5-, 10- and 20-year survival rates were 95.6%, 88.1% and 79.8%, respectively [12]. This tumour is slow-growing but has a high recurrence rate (36%), which can occur decades after initial diagnosis. Therefore, long-term follow-up is important [6]. In addition to gynaecological examination and vaginal ultrasound, AMH or inhibin can be measured during follow-up. However, the combination of AMH and inhibin B will increase the ability to detect disease recurrence [16]. In our patient, after a follow-up period of 36 months, no recurrence was detected in clinical, ultrasound and MRI as well as based on AMH levels.

## Conclusion

In conclusion, our patient is the first case of ovarian AGCT incidentally discovered during caesarean section in a pregnant patient after IVF. The relationship between ovulation induction in the treatment of infertility and ovarian cancer as well as ovarian granulosa cell tumour has not been clearly defined. Pre-operative diagnosis is difficult. Conservative surgery should be considered in women who wish to have children. Depending on the stage of the disease and prognostic factors, the treatment method is surgery, chemotherapy, radiation therapy or multimodality. Patients should receive adequate counselling and long-term follow-up to ensure the highest survival rates and early detection of recurrence.

## Abbreviation list

AGCT – adult granulosa cell tumor

## Conflicts of interest

The authors declare that they have no conflicts of interest.

## Funding

The authors received no financial support for the research, authorship and publication of this article.

## Authors’ contributions

Kong PC wrote the first draft of the paper. Nhat HM and Chi LHY collected the patient’s information. PC Kong reviewed the literature. All authors contributed to revising the manuscript and approving the final submission.

## Figures and Tables

**Table 1.** Circumstances of discovery and foetal outcomes.

Author	Patient’s age	Circumstances of discovery	Foetal outcomes	Birth weight (g)
Guidi *et al* [3]	41	Recurrent, 29 weeks	Live birth, female	2,750
Aymen *et al* [9]	30	Incidentally discovered during CS, 32 weeks gestation	Live birth	1,925
Roy and Babu [10]	23	Incidentally discovered during CS, full-term pregnancy	Live birth, male	2,500
Agarwal *et al* [11]	26	Recurrent, 20 weeks	Live birth, male	1,200
Fernández-Cid *et al* [1]	35	Tumour detected at routine ultrasound, 15 weeks' gestation	Live birth, male	NS
Current case	44	Incidentally discovered during CS, 39 weeks gestation	Live birth, male	3,000

CS, Caesarean section; NS, not stated

**Table 2.** Management and maternal outcomes.

Author	During pregnancy	Interventions during CS	After termination of pregnancy	Maternal outcomes
Guidi *et al* [3]	No	Total hysterectomy, left salpingo-oophorectomy, peritoneum biopsy	+ Six cycles with carboplatin	No recurrence after 26 months follow-up
Aymen *et al* [9]	No	Right adnexectomy and partial omentum section	+Total hysterectomy + left adnexectomy + appendicectomy + peritonium biopsy + four cycles of BEP protocol	No recurrence after 18 months follow-up
Roy and Babu [10]	NS	Right ovariectomy	Chemotherapy was used, but the authors did not specify the type of drug used	NS
Agarwal *et al* [11]	Adriamycin-Vincristine at 21 weeks	No	+Total hysterectomy + left salpingo-oophorectomy + six cycles of cisplatin	No recurrence after 10 months follow-up
Fernández-Cid *et al* [1]	NS	No	NS	Symptomatic and without recurrence on ultrasound
Current case	No	Left oophorectomy	Four-cycle regimen of Carboplatin and Paclitaxel	No recurrence after 32 months follow-up

CS, Caesarean section; NS, not stated
